# Comparison of Cardiovascular Outcomes in Patients With and Without Rheumatoid Arthritis: A Meta-Analysis of Observational Studies

**DOI:** 10.7759/cureus.40348

**Published:** 2023-06-13

**Authors:** Zineb Barkhane, Amna Zaree, Sualeha Zulfiqar, Ahmed Qudoos, Santhoshi Vaidhyula, FNU Jaiprada, Saleha Dar, Neelum Ali

**Affiliations:** 1 Medicine and Pharmacy, University of Hassan II Casablanca, Casablanca, MAR; 2 Medicine, Shalimar Medical and Dental College, Lahore, PAK; 3 Internal Medicine, Rawalpindi Medical University, Rawalpindi, PAK; 4 Medicine, Liaquat University of Medical and Health Sciences, Hyderabad, PAK; 5 Medicine, Dr. Nandamuri Taraka Rama Rao (NTR) University of Health Sciences, Vijayawada, IND; 6 Medicine, Dow University of Health Sciences, Karachi, PAK; 7 Adult Medicine, Louisiana State University Health Sciences Center, Shreveport, USA; 8 Internal Medicine, University of Health Sciences, Lahore, PAK

**Keywords:** meta-analysis, stroke, myocardial infarction, rheumatoid arthritis, cardiovascular outcomes

## Abstract

The aim of this meta-analysis was to determine the risk of incident cardiovascular disease (CVD) in patients with rheumatoid arthritis compared to patients without rheumatoid arthritis. We conducted a thorough search of online databases, including PubMed, EMBASE, and Web of Science, to identify English-language publications examining cardiovascular outcomes in patients with rheumatoid arthritis from January 1, 2005, to May 15, 2023. We followed the Preferred Reporting Items for Systematic Reviews and Meta-Analyses (PRISMA) guidelines. The search was performed using relevant keywords such as "rheumatoid arthritis," "cardiovascular diseases," and "risk," along with their synonyms. Medical subject heading (MeSH) terms and Boolean operators (AND, OR) were employed to optimize the search. Outcomes assessed in this study included composite cardiovascular events (as defined by individual studies), myocardial infarction, and stroke (including ischemic and hemorrhagic stroke). Overall, 14 studies met the inclusion criteria and were included in the present meta-analysis. We found that the risk of composite CVD was higher in patients with rheumatoid arthritis compared to patients without rheumatoid arthritis. We also found a higher risk of myocardial infarction and stroke in rheumatoid arthritis patients compared to their counterparts. This study demonstrates the elevated risk of CVD in patients with rheumatoid arthritis and highlights the importance of incorporating cardiovascular management and assessment into the care of these patients.

## Introduction and background

Rheumatoid arthritis is a chronic inflammatory illness that causes joint destruction and has a significant impact on quality of life. Chronic inflammation related to rheumatoid arthritis not only affects the joints but also the vascular system, leading to increased comorbidity and premature mortality compared to the general population, particularly from coronary artery disease (CAD) [1,2]. According to the guidelines of the European Society of Cardiology, rheumatoid arthritis is recognized as a significant risk factor for cardiovascular disease (CVD) [3]. Patients with rheumatoid arthritis have a CVD risk that is up to twice as high as the general population, nearly equivalent to the risk associated with type 2 diabetes mellitus (DM) [4]. This elevated risk of CVD is observed not only in patients with early-stage rheumatoid arthritis but also in individuals with subclinical rheumatoid arthritis (rheumatoid arthritis yet to be diagnosed). The increased CVD risk in rheumatoid arthritis cannot be solely attributed to traditional CVD risk factors or rheumatoid arthritis-related factors present at the time of diagnosis [5].

Patients with rheumatoid arthritis often experience reduced muscle mass and a low body mass index (BMI), which can be attributed to uncontrolled inflammation, limitations in physical activity, or both. In rheumatoid arthritis, having a low BMI is linked to a poorer prognosis [6]. Although cachexia, characterized by reduced muscle and fat mass, is now less common in rheumatoid arthritis, a combination of low muscle mass and high fat mass is more prevalent in rheumatoid arthritis patients. This combination can pose even greater issues concerning heart disease [7]. In rheumatoid arthritis, visceral adiposity (fat stored around the internal organs) is associated with insulin resistance, hypertension, metabolic syndrome, and an increased inflammatory burden [7].

Since the last meta-analysis comparing the CVD risk between patients with rheumatoid arthritis and patients without rheumatoid arthritis, several new studies have been conducted. Therefore, we conducted this meta-analysis to determine the risk of incident CVD in patients with rheumatoid arthritis compared to patients without rheumatoid arthritis.

## Review

Methodology

Search Strategy

We conducted a thorough search of online databases, including PubMed, EMBASE, and Web of Science, to identify English-language publications examining cardiovascular outcomes in patients with rheumatoid arthritis from January 1, 2005, to May 15, 2023. To ensure the rigor of our study, we followed the Preferred Reporting Items for Systematic Reviews and Meta-Analyses (PRISMA) guidelines. The search was performed using relevant keywords such as "rheumatoid arthritis," "cardiovascular diseases," and "risk," along with their synonyms. Medical subject heading (MeSH) terms and Boolean operators (AND, OR) were employed to optimize the search. Additionally, we manually reviewed the reference list of all included studies.

Study Selection

We included peer-reviewed cohort studies and case-control studies that met the following inclusion criteria: (a) adherence to predefined rheumatoid arthritis criteria, (b) assessment of cardiovascular events, and (c) inclusion of a comparison group. We included studies that featured patients with or without a history of CVD. We excluded studies published in languages other than English, as well as reviews, editorials, and case reports. Two investigators independently screened all eligible studies. Initial screening involved assessing titles and abstracts, followed by obtaining the full texts of eligible records for detailed assessment based on predefined inclusion and exclusion criteria. Any disagreements during the study selection process were resolved through consensus.

Data Extraction, Outcomes, and Quality Assessment

Two investigators utilized a pre-designed data extraction form in Microsoft Excel (Microsoft Corp., Redmond, WA, USA) to extract relevant data from all included studies. The extracted information included author names; year of publication; study types; sample sizes; duration of follow-up; the number of observed composite cardiovascular events (as defined by individual studies), myocardial infarction, and stroke (including ischemic and hemorrhagic stroke). Any discrepancies between the two investigators were resolved through discussion until a consensus was reached. Quality assessment of all the included studies was done using the Newcastle-Ottawa scale (NCOS).

Statistical Analysis

We employed the statistical software RevMan 5.4.1 (The Cochrane Collaboration, London, UK) to perform this meta-analysis. We calculated the risk ratio (RR) with 95% confidence intervals (CI) to compare the outcomes between patients with rheumatoid arthritis and those without rheumatoid arthritis. A significance level of p <0.05 was used to determine statistical significance. To evaluate heterogeneity among the study results, we calculated the I-square value. The choice between a random-effect or fixed-effect model was determined based on the I-square value. If the I-square value exceeded 50%, we utilized a random-effect model; otherwise, a fixed-effect model was applied.

Results

**Figure 1:**
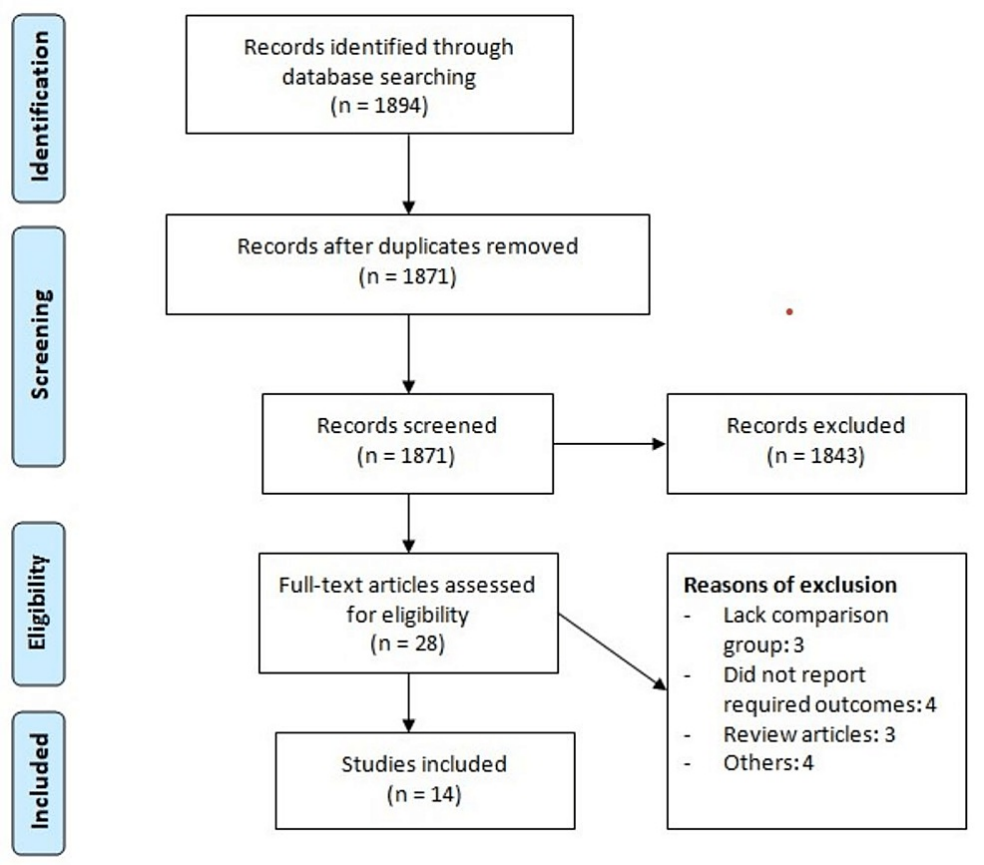
The PRISMA flowchart PRISMA: Preferred Reporting Items for Systematic Reviews and Meta-Analyses

There were 1894 studies identified through a database search. After removing duplicates, 1871 records were initially screened. Full texts of 28 studies were obtained, and on detailed assessment, 14 studies met the inclusion criteria and were included in the present meta-analysis. Figure 1 shows the process of study selection. Table 1 shows the characteristics of the included studies. The follow-up duration of the included studies ranged from one year to 13 years. Table 2 shows the quality assessment of the included studies.

**Table 1:** Characteristics of included studies RA: Rheumatoid arthritis, NR: Not reported

Authors	Year	Region	Groups	Sample Size	Follow-up	Mean age (years)	Male (%)
Ali et al. [8]	2021	Pakistan	RA	229	1 Year	46	50.2
			Non-RA	233			
Argnani et al. [9]	2021	Italy	RA	21201	5 Years	NR	NR
			Non-RA	249156			
Chung et al. [10]	2013	Taiwan	RA	29260	13 Years	52.2	23
			Non-RA	117040			
Holmqvist et al. [11]	2013	Sweden	RA	39065	3.4 Years	61	28.3
			Non-RA	171965			
Kang et al. [12]	2022	South Korea	RA	136469	4.7 Years	54.6	26.4
			Non-RA	682345			
Lai et al. [13]	2020	Taiwan	RA	748	2.7 Years	70.5	68.5
			Non-RA	189922			
Lee et al. [14]	2021	South Korea	RA	2765	12 Years	53.5	26.6
			Non-RA	13825			
Lindhardsen et al. [15]	2011	Denmark	RA	9921	4.6 Years	51.3	48.5
			Non-RA	3978821			
Maradit-Kremers et al. [16]	2005	United States	RA	603	2 Years	58.1	26.9
			Non-RA	603			
Myasoedova et al. [17]	2021	United States	RA	905	4.2 Years	55.9	31.4
			Non-RA	904			
Nikiphorou et al. [18]	2020	United Kingdom	RA	6591	5.4 Years	58.7	32.5
			Non-RA	6591			
Peters et al. [19]	2009	Netherland	RA	312	3 Years	62.5	45.2
			Non-RA	1852			
Pujades-Rodriguez et al. [20]	2016	England	RA	12120	4.2 Years	56.5	27.7
			Non-RA	121191			
Solomon et al. [21]	2006	United States	RA	25385	5 Years	NR	29.1
			Non-RA	252976			

**Table 2:** Quality assessment of included studies

Authors	Selection	Comparison	Outcome	Overall
Ali et al. [8]	2	2	2	Fair
Argnani et al. [9]	3	2	3	Good
Chung et al. [10]	3	2	3	Good
Holmqvist et al. [11]	4	2	3	Good
Kang et al. [12]	3	2	3	Good
Lai et al. [13]	3	1	2	Fair
Lee et al. [14]	2	2	2	Fair
Lindhardsen et al. [15]	3	2	3	Good
Maradit-Kremers et al. [16]	4	2	3	Good
Myasoedova et al. [17]	3	2	3	Good
Nikiphorou et al. [18]	4	2	3	Good
Peters et al. [19]	2	2	2	Fair
Pujades-Rodriguez et al. [20]	4	2	2	Good
Solomon et al. [21]	3	1	2	Fair

Meta-Analysis of Outcomes

**Figure 2:**
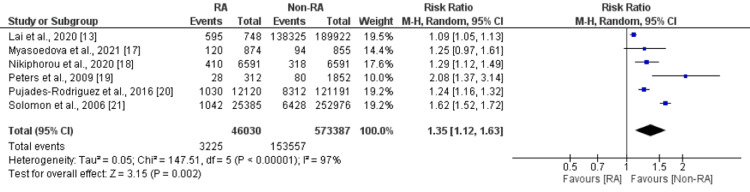
Forest plot comparing CVD between RA and non-RA patients CVD: Cardiovascular disease, RA: Rheumatoid arthritis

**Figure 3:**
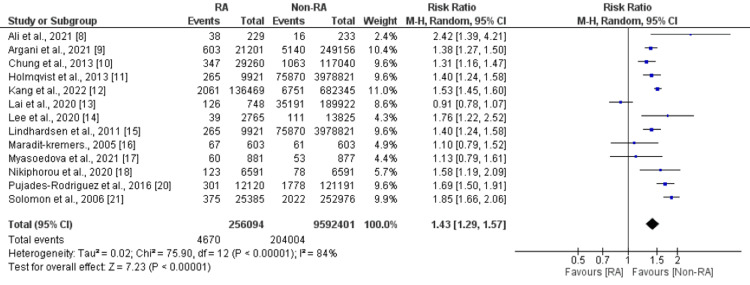
Forest plot comparing myocardial infarction between RA and non-RA patients RA: Rheumatoid arthritis

**Figure 4:**
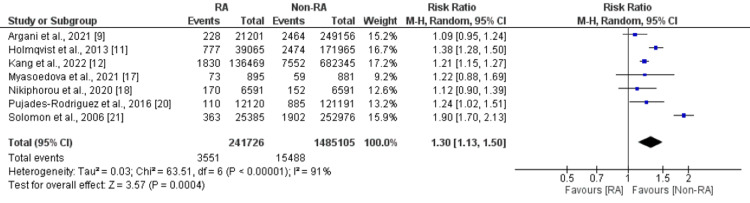
Forest plot comparing stroke between RA and non-RA patients RA: Rheumatoid arthritis

Six studies compared the risk of CVD between patients with rheumatoid arthritis and patients without rheumatoid arthritis. A pooled analysis of six studies reported that the risk of developing CVD was 1.35 times significantly higher in patients with rheumatoid arthritis compared to their counterparts (RR: 1.35, 95% CI: 1.12-1.63, p=0.002) as shown in Figure 2. High heterogeneity was reported among the study results (I-square: 97%). Thirteen studies were included in the pooled analysis of the comparison of myocardial infarction between patients with rheumatoid arthritis and patients without rheumatoid arthritis. As shown in Figure 3, the risk of developing myocardial infarction was significantly higher in patients with rheumatoid arthritis (RR: 1.43, 95% CI: 1.29-1.57, p <0.001). High heterogeneity was reported among the study results (I-square: 84%). Seven studies were included in the pooled analysis of the risk of stroke. As shown in Figure 4, the risk of stroke was 1.30 times higher in patients with rheumatoid arthritis compared to their counterparts. High heterogeneity was reported among the study results (I-square: 91%).

Meta-Regression

To explore the heterogeneity, we performed a meta-regression analysis to examine the association between certain variables (age, male, diabetes, hypertension, and BMI) and three outcomes (CVD, myocardial infarction, and stroke). Table 3 showcases the results (in the form of p-values) that showed having diabetes was a statistically significant predictor of an increased risk of CVD and myocardial infarction.

**Table 3:** Results of meta-regression CVD: Cardiovascular disease; BMI: Body mass index * significant at p <0.05

Variable	CVD (p-value)	Myocardial infarction (p-value)	Stroke (p-value)
Age	0.057	0.069	0.48
Male	0.13	0.25	0.33
BMI	0.33	0.28	0.39
Diabetes	0.046*	0.032*	0.058
Hypertension	0.22	0.13	0.039*

Discussion

This study aims to assess the risk of CVD in patients with rheumatoid arthritis. We found that the risk of composite CVD was higher in patients with rheumatoid arthritis compared to patients without rheumatoid arthritis. We also found a higher risk of myocardial infarction and stroke in rheumatoid arthritis patients compared to their counterparts.

Seven studies compared the risk of stroke in patients with rheumatoid arthritis and patients without rheumatoid arthritis. In all studies, the risk was found to be greater in patients with rheumatoid arthritis. The meta-analysis conducted by Avina-Zubieta et al. identified seven studies featuring 39,520 patients with rheumatoid arthritis, who were assessed for the risk of stroke. The study reported a 41% increase in the risk of stroke in patients with rheumatoid arthritis [22].

Systemic inflammation plays a crucial role in the development of CVD. It affects various other CVD risk factors, leading to a unique association between cardiovascular risk and rheumatoid arthritis compared to the general population [23]. The pro-inflammatory cytokines involved in rheumatoid arthritis, such as tumor necrosis factor-alpha (TNF-a) and interleukin-6 (IL-6), contribute to the development of atherosclerosis by directly damaging the endothelium of blood vessels. They also interfere with the vascular repair system and modulate classic risk factors. Inflammation, both innate and adaptive, influences the initiation, progression, and destabilization of atherosclerosis [24,25]. A clinical study showed that after receiving an infliximab infusion, there was an increase in the percentage of cases exhibiting a temporary improvement in endothelial function (endothelial-dependent vasodilation). This suggests that long-term TNF blockade reduces the incidence of cardiovascular complications in rheumatoid arthritis [26]. This finding aligns with previous research that has demonstrated a relationship between disease severity and the occurrence of CVD [27,28].

While the current European Alliance of Associations for Rheumatology (EULAR) and European Resuscitation Council (ERC) guidelines identify the significance of adequate clinical management of patients with rheumatoid arthritis to prevent CVD, the equivalent United States guidelines, including the American College of Cardiology and American Heart Association (ACC/AHA) recommendations, do not specify the need for cardiovascular management and assessment [29,30]. Additionally, no interventional studies have been carried out to assess the efficiency of executing primary prevention therapy and monitoring targets for patients with rheumatoid arthritis. The study findings support the requirement for this type of study. Similarly, there is evidence indicating a connection between rheumatoid arthritis and the subsequent occurrence of certain cardiovascular conditions. However, there is a lack of data related to the relationship between rheumatoid arthritis and other conditions like congestive heart failure in the general population. This finding holds significant implications for risk assessment because the existing recommended risk scores for RA patients have been developed based on angina, acute myocardial infarction, and cerebrovascular endpoints [31].

The present meta-analysis has certain limitations. We included studies with different clinical settings, diagnostic criteria, age at enrollment, study design, and period at risk. We found statistically significant heterogeneity among the study results. As recommended, we utilized the random-effects model to deal with variability. We also performed meta-regression to explore variables affecting it. Furthermore, we lacked patient-level data to explore the effect of rheumatoid arthritis on different subgroups, including patients with and without diabetes, hypertension, and so on.

## Conclusions

This meta-analysis demonstrates that patients with rheumatoid arthritis are at a higher risk of developing CVD compared to those without rheumatoid arthritis. The findings indicate a significantly increased risk of composite CVD, myocardial infarction, and stroke in these patients. This study also highlights the importance of incorporating cardiovascular management and assessment into the care of these patients. Further research is warranted to address the identified gaps and improve risk assessment strategies for patients suffering from rheumatoid arthritis, considering a broader range of cardiovascular conditions.
